# Whole-slide imaging and a Fiji-based image analysis workflow of immunohistochemistry staining of pancreatic islets

**DOI:** 10.1016/j.mex.2022.101856

**Published:** 2022-09-13

**Authors:** Emma Jane Buckels, Jacqueline Mary Ross, Hui Hui Phua, Frank Harry Bloomfield, Anne Louise Jaquiery

**Affiliations:** a Liggins Institute, University of Auckland, Auckland, New Zealand; b Department of Anatomy and Medical Imaging, University of Auckland, Auckland, New Zealand

**Keywords:** β-cell mass, α-cell mass, Semi-automated image analysis, Type 2 diabetes mellitus, Sheep pancreas

## Abstract

Quantification of cell populations in tissue sections is frequently examined in studies of human disease. However, traditional manual imaging of sections stained with immunohistochemistry is laborious, time-consuming, and often assesses fields of view rather than the whole tissue section. The analysis is usually manual or utilises expensive proprietary image analysis platforms. Whole-slide imaging allows rapid automated visualisation of entire tissue sections. This approach increases the quantum of data generated per slide, decreases user time compared to manual microscopy, and reduces selection bias. However, such large data sets mean that manual image analysis is no longer practicable, requiring an automated process. In the case of diabetes, the contribution of various pancreatic endocrine cell populations is often investigated in preclinical and clinical samples. We developed a two-part method to measure pancreatic endocrine cell mass, firstly describing imaging using an automated slide-scanner, and secondly, the analysis of the resulting large image data sets using the open-source software, Fiji, which is freely available to all researchers and has cross-platform compatibility. This protocol is highly versatile and may be applied either in full or in part to analysis of IHC images created using other imaging platforms and/or the analysis of other tissues and cell markers.

## Graphical abstract

**Figure:**
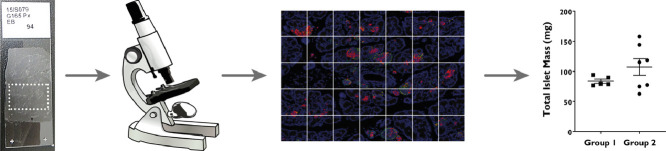

**Specifications table**

**Table:** 

Subject Area	Biochemistry, Genetics and Molecular Biology
More specific subject area	Immunohistochemistry and image analysis
Protocol name	Whole-slide imaging and image analysis workflow of pancreas sections.
Reagents/tools	**Microscope** • MetaSystems VSlide slide-scanner (MetaSystems, Altlussheim, Germany. This system consisted of: ▪ Microscope: Zeiss AxioImager Z2 microscope (Carl Zeiss Microscopy, Jena, Germany). ▪ Camera: monochrome CoolCube 1m camera (MetaSystems, Altlussheim, Germany). ▪ Stage: motorised five-position scanning stage (Marzhauser, Wetzlar, Germany). ▪ SlideFeeder x80 (MetaSystems, Altlussheim, Germany). ▪ A quad filter (filter set 81 HE; Carl Zeiss Microscopy, Jena, Germany) in conjunction with a Colibri.2 (Carl Zeiss Microscopy, Jena, Germany) light source to visualise Alexa Fluor 488, Alexa Fluor 647, and DAPI fluorophores. ▪ A filter specific to Alexa Fluor 594 (filter set 45 HQ TexasRed; Carl Zeiss Microscopy, Jena, Germany) in conjunction with an X-Cite 120PC Q (Excelitas Technologies, Waltham, Massachusetts, USA) light source to visualise the Alexa Fluor 594 fluorophore. **Software:** • Metafer software (version 3.12.6; MetaSystems, Altlussheim, Germany) to control light sources, filter sets, and exposure times for each assay. • VSlide software (version 1.1.107; MetaSystems, Altlussheim, Germany) to stitch raw images into one whole-slide image. • MetaViewer software (version 2.0.133; Metasystems, Altlussheim, Germany) to view stitched whole-slide images. • Fiji (version 1.53g; Fiji Is Just ImageJ, NIH, Maryland, USA) for subsequent image analysis.
Experimental design	Pancreata were removed from 132-133 days gestational age fetal lambs at *post mortem* examination. Pancreata were sectioned into two halves along the midline of the organ so that each half contained head, body, and tail regions. One half was preserved in neutral buffered 10% formalin and transferred into 70% ethanol after 72-hours for storage until processing. The other half was snap-frozen in liquid nitrogen for separate molecular biology experiments. Formalin-fixed pancreata were paraffin-embedded, and 5 µm thick sections were cut and placed onto SuperFrost plus adhesion slides (Thermo Fisher Scientific). Five tissue sections per lamb, at least 100 µm apart, underwent multiplexed immunohistochemistry (IHC) for insulin, glucagon, and somatostatin. A MetaSystems VSlide slide-scanner (MetaSystems) was used to image stained tissue sections. We acquired approximately 450-800 fields of view (FOV) per slide using a Plan Apochromat 20x/0.8 NA objective lens. Image analysis was performed on individual FOV images using Fiji (NIH, USA), an open-source image analysis software. We used the following image analysis workflow comprising a sequence of 2 macros: [1] image cropping and file renaming (macro 01: cropping and renaming macro); [2] image analysis, which included defining regions without tissue, identifying and measuring insulin-positive, glucagon-positive, and somatostatin-positive staining (macro 02: analysis macro); and followed by [3] image quality control.
Trial registration	Not applicable.
Ethics	Experimental protocols which generated pancreata used within this protocol were approved by The University of Auckland Animal Ethics Committee (approval number R001101).
Value of the Protocol	Identifying and quantifying the percentage of different cell types in tissues is important in many studies of human disease. Our protocol describes the imaging of specific labelling for various endocrine cell populations in paraffin-embedded pancreata sections from sheep using whole-slide imaging and a workflow to analyse these image-sets. • Whole-slide imaging enables rapid visualisation of entire tissue sections in an automated process without the need to employ random sampling, which is often not possible with available instrumentation. Many tissues contain multiple components, and traditional methods of analysing these rely on adopting a random approach to selecting a limited number of fields of view. For example, the endocrine pancreas comprises the cells of interest but only makes up 2-5% of the total pancreas. Our approach is comprehensive and increases the amount of data-per slide generated, thereby decreasing the likelihood of sampling bias compared to the usual practice of imaging small regions from each section. Whole-slide imaging also significantly reduces hands-on time compared to traditional manual microscopy. • We designed an image analysis workflow to analyse the large data sets typically generated from whole-slide imaging, using software freely available to researchers. Generally, image analysis of this nature is performed manually, which is not practicable for image data sets of this size, or by using proprietary software that is often expensive and where the vendor is not able to fully disclose the details of the steps involved in the analysis. Our image analysis workflow uses the open-source image analysis platform, FIJI, with all code fully described within this protocol, allowing for full transparency of the processes involved with image analysis. This full disclosure will enable others to adapt our method to suit their image data if required. • This protocol can be applied to samples from other species and other tissues and cell markers where quantifying areas of positive staining are required. Therefore, whilst we have applied this method to the ovine endocrine pancreas, this protocol is flexible, so it can be adapted as required by the researcher. We have already applied the method to murine endocrine pancreas samples. • This image analysis workflow can easily be adapted to analyse images from different microscope platforms that generate whole-slide images. After making minor changes to Macro 01, we used the image analysis workflow for an image data set generated on another microscope platform.

## Description of protocol

### Background

Quantification of specific cell types in tissues provides valuable information for studies of human diseases, such as type 2 diabetes mellitus (T2DM). Measurements of pancreatic endocrine cell mass, such as β-cell mass, are frequently carried out in animal models of islet biology and type 2 diabetes mellitus (T2DM) due to the relationship between β-cell mass and β-cell function [1], [2], [3], [4]. Tissue sections from sheep pancreata that underwent multiplexed immunohistochemistry (IHC) using previously validated antibodies [5], [6], [7], [8], [9] were used to develop this protocol. Sheep are used as a paradigm for examining the effects of the early life environment on offspring as they typically have singleton pregnancies, and lambs and humans are at a similar stage of organ development at birth [10].

The protocol is comprised of two parts; image acquisition and analysis. First, we describe whole-slide imaging of paraffin-embedded tissue sections. By acquiring an image of the entire tissue section, any decisions over the merits of systematic or random sampling can be avoided. Although whole-slide imaging technology has been available in some form since the late 1990s, limiting the imaging of IHC staining to approximately 20 fields of view (FOV) across five different tissue sections throughout the pancreas is still prevalent in many contemporary publications. Whereas in the current study, imaging the entire tissue section provides 400-600 FOV. Image acquisition is followed by a semi-automated image analysis workflow, which we developed to analyse image data sets generated by whole-slide imaging. The workflow was specifically designed to analyse the large data sets generated from whole-slide imaging. The value of this protocol lies within the increased accuracy and precision of measuring cell populations using whole-slide imaging compared to traditional manual microscopy, without the need for high-performance computing or proprietary software. While the entire section is imaged and stitched, the analysis is carried out using individual FOV, which reduces the need for very high computing power as the memory and processor requirements are less than if the full resolution stitched image was analysed.

All code is included, allowing complete transparency of the analysis methods. This code was written for Fiji, a free, open-source software available for all platforms [11]; however, other open-source software programs are also available, including QuPath, Orbit Image Analysis, and PathML. Whilst we have applied our method to measuring endocrine cell populations of the pancreas, this full disclosure will enable others to adapt our method to suit their needs and to measure the area of other specific cell populations.

### Experimental procedures

To generate the slides used for this protocol, 19 formalin-fixed ovine pancreata were paraffin-embedded, and 5 µm thick sections were cut and placed onto SuperFrost plus adhesion slides (Thermo Fisher Scientific). Five tissue sections per lamb, at least 100 µm apart, underwent multiplexed immunohistochemistry (IHC) for insulin, glucagon, and somatostatin [9]. A detailed description of this methodology can be found in Supplementary Method Note 1.

### 1. Imaging

We used a MetaSystems VSlide slide-scanner to image our slides. However, other microscope platforms that generate whole-slide images can be used.

1.1. Clean slides with 70% ethanol wipes. Place the slides into the microscope trays and load these trays into the SlideFeeder. Up to 80 slides can be loaded into the SlideFeeder at a time.
1.2. Determine exposure settings for each fluorophore. Exposure settings should be modified to ensure maximal signal and minimal background. Determine appropriate exposure settings at the start of each scanning session to ensure that images are not over-saturated, as this can affect the subsequent analysis. These values may need to be adjusted for different staining runs.
1.3. Perform a pre-scan of the slides at 2.5x magnification to create a focus map for the section using the DAPI channel. This focus map establishes the contour of the section on the slide and is used to automatically adjust the focus position across the section when the system scans the slide at higher magnification. This also identifies the location of tissue areas based on DAPI staining, as all cell nuclei in the tissue will be stained.
  1.3.1. From this pre-scan, confirm the tissue region of interest (ROI) to be scanned at 20x magnification. The ROI will contain approximately 400-600 FOV per slide depending on the size of the section and should include approximately equal portions of the head, body, and tail regions of each pancreas.
  1.3.2. Scan the selected ROI at 20x magnification
1.4. The VSlide software stitches the raw images into one virtual slide image. Stitched images can be viewed offline using MetaViewer software.
1.5. Raw images from each FOV (one image per fluorophore filter per FOV, in TIFF format) are automatically saved in addition to the software-generated stitched image (one per slide, saved in Visual Studio Community Content Installer (VSI) format) for each slide scanned. Raw images from each FOV are acquired with a proportion of overlap with neighbouring FOV.
  Note: if using a platform other than the MetaSystems VSlide slide-scanner, ensure both the raw and stitched images are saved.
1.6. Store images in a secure location with automatic daily backup to ensure no data is lost.

### 2. Image analysis

Perform image analysis using Fiji [11] software as described in the workflow below. Use the same version of Fiji for all analyses. All macros are written in ImageJ1Macro (IJM) programming language. All image analysis is carried out on individual FOV images. We perform image analysis on individual FOV images as whole-slide imaging often creates image files in excess of 2 GB each, introducing a requirement for high-powered computers. However, the use of individual FOV to perform image analysis circumnavigates the requirement for significant amounts of computer memory. Save files with the file hierarchy and format described in Table 1. Note: images generated from microscope platforms other than the MetaSystems VSlide slide-scanner can be analysed using this workflow. The only requirements are that the slide file name and slide ID follow the naming hierarchy as described in Table 1. If the classifier file extension is different, macro “02 Analysis macro.ijm” can be amended to include the appropriate extension (lines 93, 97, 101, and 105).

**Table 1:** Slide ID naming hierarchy and file hierarchy levels required for use with this protocol.

Slide ID			
Category	Format	Example	File Hierarchy Level
User name initials followed by an underscore	XX_	EB_	N/A
Date of acquisition	YYMMDD	160117	N/A
Project ID	XXXX	Sild	1
Protocol ID (insulin, glucagon, somatostatin)	IGS	IGS	2
Slide Run Number	Slide XX	Slide 10	3
Animal ID	XXXXXX	15S094	4
Full file name for slide before acquisition	XX_YYMMDD XXXX IGS Slide XX XXXXXX	EB_160117 Sild IGS Slide 10 15S094	N/A

Additions to Slide file name during acquisition			

Category	Format	Example	File Hierarchy Level

FOV (numbered sequentially - 6 digits)	Img-XXXXXX-	Img-0000001-	N/A
Classifier (B, Y, G, O)	X	B	N/A

A description of the required image naming hierarchy, with examples of file and image names that follow the convention.

The naming convention for each slide was: user initials (e.g. EB), date of scan (YYMMDD, e.g. 160117), a four-letter project code (our project code was “Sild”), protocol ID (IGS; representing insulin, glucagon, somatostatin), Slide Run Number (for our project, these ranged from 10-90), and Animal ID (a six-digit code unique to each animal which can be a mixture of numbers and characters). During image acquisition, a FOV ID is added, which is the specific code for each FOV image. One image is generated for each fluorophore used, with the colour specified by the last character (B; DAPI staining, Y; insulin staining. G; glucagon staining, O; somatostatin staining). This is based on the classifiers used by the MetaSystems VSlide. As there is no information for the Animal ID or Slide ID in the FOV ID, macro 01 appends the correct Slide Number and Animal ID to each FOV ID.

**Fig. 1:**
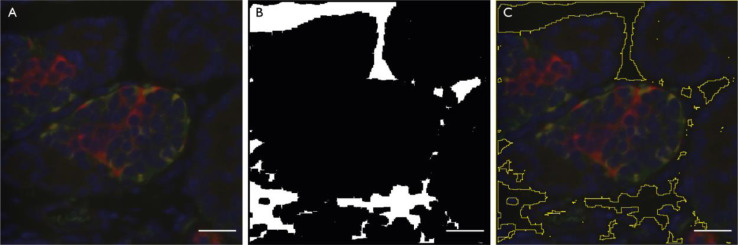
Representative images from the process of defining regions without tissue in images of pancreatic tissue. In this process, images from all four channels per FOV are pseudo-coloured and merged into one composite image (A). This composite image is converted into binary to identify and measure regions with no tissue. These regions are overlaid onto the binary image (B) and the composite image (C) and saved, allowing the user to subsequently confirm that the macro has worked as intended during image quality control. The scale bar represents 25 µm.
  2.8.2. It will open each image taken for insulin, glucagon, and somatostatin immunofluorescence and measure regions of positive staining greater than the background and above a user-specified size threshold to exclude debris (Fig. 2).

2.1. Open Fiji macro “01 Cropping and renaming macro.ijm”.
2.2. Confirm the region of interest for cropping (line 72) based on the areas of overlap between neighbouring FOV. This region of interest will be applied by macro 01 to all images in the data set. It will uniformly crop the images to remove areas that are also present in neighbouring images.
2.3. Run macro “01 Cropping and renaming macro.ijm”. The pipeline is detailed below.
  2.3.1. A duplicate of each original image is created and used for the analysis. This ensures that the integrity of the image data set is maintained.
  2.3.2. The image is cropped according to 2.2 above.
  2.3.3. A new folder for each slideID is created with the added suffix “Crop” has been generated, and each image is renamed and saved in this folder.
2.4. Open macro “02 Analysis macro.ijm”.
2.5. Confirm the calibration for the system (line 116). For our objective lens and camera, this was 3.1 pixels per µm.
2.6. Confirm the threshold for background tissue calculation (line 170).
2.7. Confirm the threshold values for insulin (line 200), glucagon (line 226), and somatostatin (line 253) positive staining regions. These values may need to be adjusted for different rounds of staining.
2.8. Run macro “02 Analysis macro.ijm”. The pipeline is detailed below.
  2.8.1. This macro first defines the regions where there is no pancreatic tissue (Fig. 1).

**Fig. 2:**
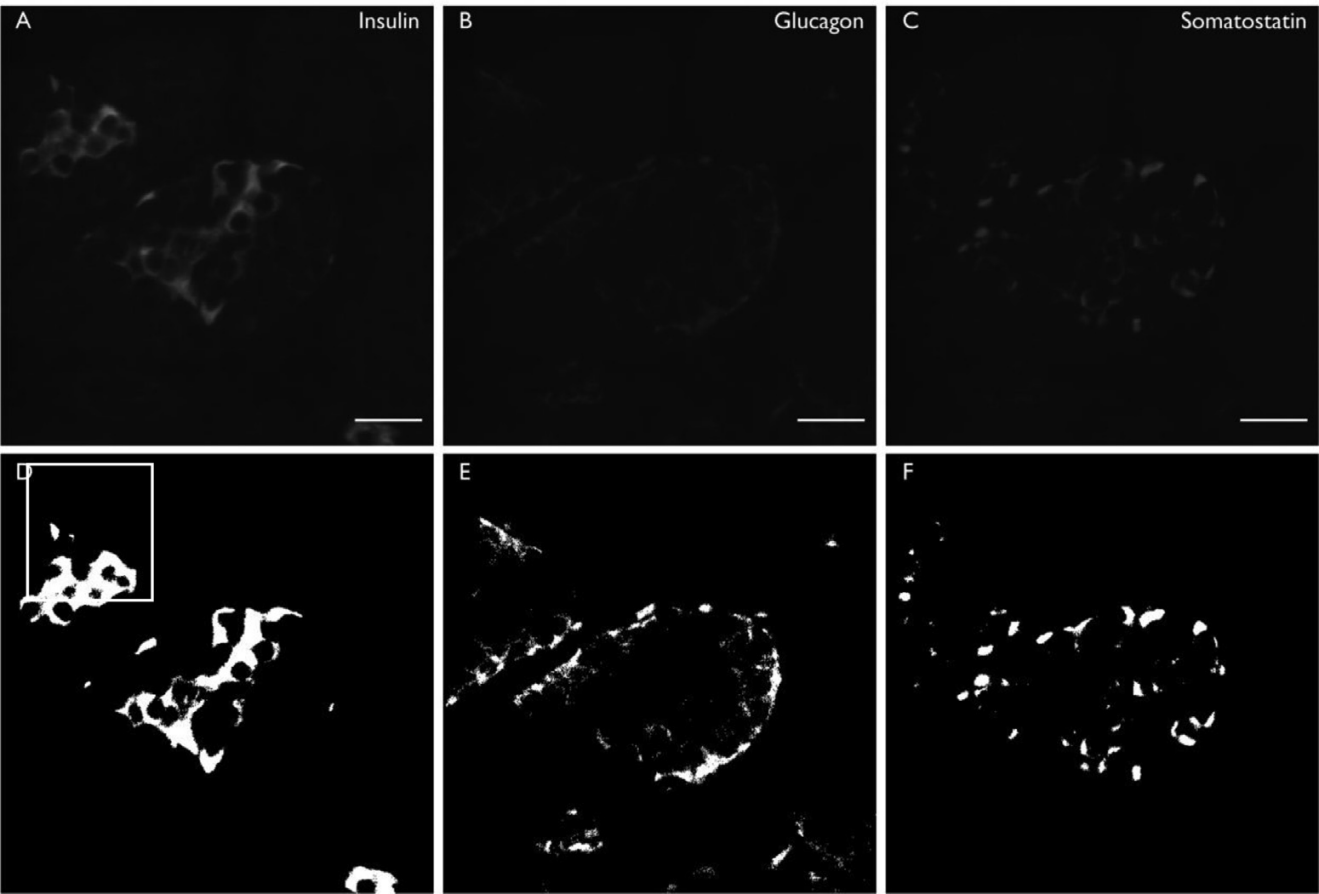
Representative images showing immunofluorescence staining of pancreatic endocrine cells (A-C) and the measured regions (D-F). First, the cropped insulin image (A) is opened, a threshold determined by the user is applied, the image is converted into binary, and regions where there is insulin-positive staining are measured. These regions are overlaid onto the binary image (D), allowing the user to subsequently confirm that the macro has worked correctly during image quality control. This process is repeated for glucagon images (B and E, respectively) and somatostatin images (C and F, respectively). The scale bar represents 25 µm.
  2.8.3. A new folder is created within each slideID called “outputs”. The folder will contain seven images per FOV and a summary text file.
2.9. Perform image quality control following the completion of image analysis.
  2.9.1. For each slide, compare the output images from approximately 10 FOV to the raw images for each fluorophore to ensure that the threshold values selected were appropriate and, therefore, that the analyses were executed correctly and generated accurate results for each FOV. If any of the results from this subset of images are not as expected, adjust the threshold value and repeat the analysis.
    Note that the appropriate threshold values must be determined experimentally by the user and validated using manual image analysis, as staining intensity can vary between laboratories.
  2.9.2. Examine the whole section stitched images using MetaViewer (or the corresponding viewing software if a different microscope platform was used) to identify any staining artefacts present, such as tissue folding, focus issues, or excessive/inappropriate background staining that might confound the results. If such artefacts are detected, exclude the identified FOV from further analyses.
2.10. Pool results.
  Use Power Query in Excel to combine data from multiple summary files into a single table, based on the column headers non-tissue area, total image area, insulin-positive area, glucagon-positive area, and somatostatin-positive area. The process is described below.
  2.10.1. Using Power Query in Microsoft Excel, open each summary text file into one large table.
  2.10.2. Remove any identified FOV with artefacts from the summary table.
  2.10.3. Using Power Pivot, calculate the sum of the background area, total area, insulin-positive area, glucagon-positive area, and somatostatin-positive area for each slide ID.
  2.10.4. Using Power Pivot, calculate the average for total background area, total area, insulin-positive area, glucagon-positive area, and somatostatin-positive for each animal ID.
    A results table for each animal ID is created, including total background area, total area scanned, total insulin-positive staining area, glucagon-positive staining area, and somatostatin-positive staining area. All measurements are in µm^2^.

## 3. Endocrine Cell Mass Calculations

3.1 Calculate total tissue area (µm^2^) by subtracting the total background area (µm^2^) from the total area scanned (µm^2^).
3.2 Calculate the β-cell mass using the following formula, modified from Limesand et al. (5);
  $$\beta \text{-}\text{cell}\;\text{mass}\;\left(\text{mg}\right)=\frac{\text{insulin}\text{-}\text{positive}\;\text{area}\;\left(\mu m^{2}\right)\times \text{pancreas}\;\text{weight}\;\left(\text{mg}\right)}{\text{total}\;\text{tissue}\;\text{area}\;(\mu m^{2})}$$
3.3 Repeat for calculations of α- and δ-cell mass, substituting glucagon-positive and the somatostatin-positive areas, respectively.
3.4 Calculate endocrine cell masses as above for individual slide runs to check whether any individual slides are artificially distorting the generated data for each animal. This check will prompt the user to investigate these slides further if results between slide runs are highly variable (e.g. was the staining particularly strong with one of the five slides?).
3.5 Perform an outlier analysis using the ROUT method of identifying outliers in Prism (Prism for Windows, version 7.03; GraphPad Software, California, USA).
3.6 Calculate total islet cell mass by adding together the cell mass values calculated for β-, α-, and δ-cells.
3.5 Calculate the percentage of each islet cell type with respect to total islet cell mass by dividing the values calculated for β-, α-, and δ-cell mass by the total islet cell mass, multiplied by 100.

### Type II ANOVA

Traditional manual imaging of stained tissue sections relies on the microscope user adopting a systematic sampling approach to selecting a predefined number of FOV to image per slide. Random sampling is not possible with manual image acquisition. This predefined number is commonly 20 FOV, and this practice remains in common use today. However, it is challenging to ensure that the fields selected accurately represent the population of cells on the slide. Further, the practice of imaging small regions from each section increases the risk of sampling bias. This is exacerbated if higher magnification objective lenses are used as the fields of view are smaller.

We used Type II ANOVA to assess the sources of variation in our endocrine cell mass measurements. This model measures three sources of variation: the total variation from every FOV on all five slides (the total sum of squares); the variation between every FOV for each slide (the sum of squares within), and the variation between slides (the sum of squares between). We performed this analysis for the insulin marker. To account for FOV, which included non-tissue areas, we subtracted total tissue area minus background tissue area for each FOV and calculated insulin-positive areas as a percentage of total tissue area per FOV.

**Fig. 3:**
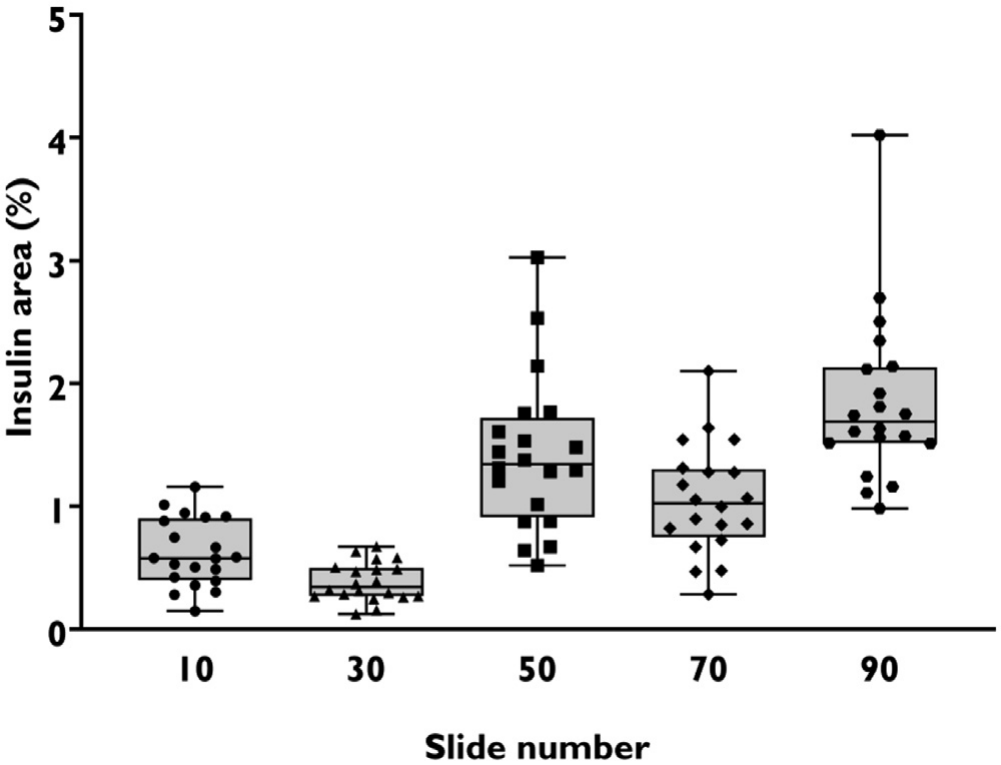
Mean insulin area of sampling events generated during simulated systematic random sampling of slides from animal 15S097. Each symbol in the boxplot represents the mean of the 20 randomly selected FOV from each slide during the process used to simulate systematic random sampling. There is an equal chance of each mean value being selected when a systematic random sampling approach is used. Lower and upper box boundaries represent 25^th^ and 75^th^ quartiles, and the upper and lower error lines represent minimum and maximum values.

In our first Type II ANOVA, we used the dataset generated from whole-slide imaging to simulate a random sampling approach to selecting FOV. For this, we used a random number generator to select one animal. We then used another random number generator to select 20 FOV from each of the five slides from that animal. This process was repeated 20 times, which provided a total of 2000 data points (Fig. 3). We entered these 2000 data points into our Type II ANOVA. We found that the largest source of variation was within FOV on each slide in 17 out of 20 random samples.

In our second Type II ANOVA, we used the entire dataset generated from whole-slide imaging. Each animal had a total of 2375 ± 141 FOV across all five slides per animal. We applied the model to samples from 19 animals. This time we found that the largest source of variation was between slides in 17 out of 19 animals. This indicates that with the large number of sampling events per slide, variation between FOV on each slide was minimal compared to the variation that arose from sampling the tissue at various depths.

Thus, increasing the number of FOV measured per slide in whole-slide imaging dramatically decreases the variation between sampling events on each slide compared to a systematic but limited approach to selecting FOV. Increasing the number of FOV imaged increases the accuracy (values reported reflect the actual value for a measurement) and the precision (the degree to which the same results can be obtained when the measurement is repeated numerous times) of quantifying cell populations in tissue sections.

## Declaration of interests

The authors declare that they have no known competing financial interests or personal relationships that could have appeared to influence the work reported in this paper.

## Appendix Supplementary materials

Image, application 1

Image, application 2

Image, application 3
