# Supplementary material for: Whole-slide imaging and a Fiji-based image analysis workflow of immunohistochemistry staining of pancreatic islets
Source: MethodsX. 2022 Sep 13;9:101856. doi: 10.1016/j.mex.2022.101856 (PMC9531276; doi:10.1016/j.mex.2022.101856)
Supplement: Supplementary file 1 [file mmc1.docx]

**Supplementary Method Note 1 - Immunohistochemistry (IHC) staining of β-, α-, and δ-cells in paraffin-embedded pancreas sections**

**Reagents**

Neutral buffered 10% formalin (Sigma Aldrich, Missouri, USA).

Ethanol (Merck Millipore, Darmstadt, Germany). Dilute ethanol with dH2O when required.

Xylene (VWR, Pennsylvania, USA).

Paraplast embedding media paraffin wax (Leica Biosystems, Wetzlar, Germany).

1x phosphate-buffered saline (1x PBS; 137 mM NaCl, 2.7 mM KCl, 10 mM Na2HPO4, 1.8 mM KH2PO4, pH 7.4).

10 mM Sodium citrate buffer (10 mM sodium citrate acid (Sigma Aldrich, Missouri, USA), 0.05% TWEEN 20 (Sigma Aldrich, Missouri, USA), in dH2O, pH 6.0).

Bovine serum albumin (BSA) blocking buffer (2% w/vol BSA (NZ origin, low IgG grade; MP Biomedicals NZ, Auckland, New Zealand) in 1x PBS).

A hydrophobic barrier pen (Vector Laboratories, Burlingame, California, USA).

Goat serum blocking buffer (5% vol/vol normal goat serum (NZ origin; Gibco, Thermo Fisher Scientific, Massachusetts, USA), 2% w/vol BSA (NZ origin, low IgG grade; MP Biomedicals NZ, Auckland, New Zealand), 0.05% w/vol sodium azide (Sigma Aldrich, Missouri, USA), 0.01% vol/vol Triton X-100 (Sigma Aldrich, Missouri, USA), 0.05% TWEEN 20 (Sigma Aldrich, Missouri, USA). In 1x PBS). Note: we created a 10x stock containing 20% w/vol BSA and 0.5% w/vol sodium azide for use with this buffer and the primary antibodies cocktail.

6-diamidino-2-phenylindole (DAPI; Sigma Aldrich, St Louis, Missouri, USA), diluted to 0.5 µg/mL in 1x PBS.

ProLong Gold Antifade Mountant (Thermo Fisher Scientific, Massachusetts, USA).

**Antibodies**

Guinea pig anti-porcine insulin (catalogue number DAKOA056401, DAKO, Glostrup, Denmark).

Mouse anti-porcine glucagon (catalogue number G2654.5mL, Sigma Aldrich, Missouri, USA).

Rabbit anti-human somatostatin (catalogue number DAKOA056601, DAKO, Glostrup, Denmark).

Goat anti-Guinea Pig IgG (H+L) Highly Cross-Adsorbed Secondary Antibody, Alexa Fluor 647 (catalogue number A-21450, Invitrogen, Thermo Fisher Scientific, Massachusetts, USA).

Goat anti-Mouse IgG (H+L) Highly Cross-Adsorbed Secondary Antibody, Alexa Fluor 488 (catalogue number A-11029, Invitrogen, Thermo Fisher Scientific, Massachusetts, USA).

Goat anti-Rabbit IgG (H+L) Highly Cross-Adsorbed Secondary Antibody, Alexa Fluor 594 (catalogue number A-11037, Invitrogen, Thermo Fisher Scientific, Massachusetts, USA).

**Equipment**

Tissue embedding cassettes (Techno Plas, Adelaide, Australia).

Automated tissue processing machine (Leica Biosystems, Wetzlar, Germany).

Heated paraffin embedding module (Leica Biosystems, Wetzlar, Germany).

Jung Biocut 2035 Microtome (Leica Biosystems, Wetzlar, Germany).

Water bath for paraffin sections (Leica Biosystems, Wetzlar, Germany).

S35 fine microtome blades (Feather Safety Razor Co, Osaka, Japan).

SuperFrost Plus adhesion microscope slides (Thermo Fisher Scientific, Massachusetts, USA).

2100 Antigen Retriever (Prestige Medical, Lancashire, UK).

Coverslips (Thermo Fisher Scientific, Massachusetts, USA).

**Protocol**

**1. Tissue Collection**

Collect tissue from fetal lambs (132-133 days gestational age, term = 148 days gestational age) as detailed below.

1.1 Inject ewes with 100-120 mg.kg-1 sodium pentobarbitone (300 mg.mL-1, Provet, Auckland, New Zealand) into the jugular vein. Once consciousness is lost, exsanguinate animals. Remove the uterus and open it. If a pulse is detected on the fetal lamb, inject the lamb with sodium pentobarbitone intravenously via either the umbilical vein or the jugular vein.

1.2 Identify and remove the pancreas, and section it into two halves along the midline of the organ. Each half should contain head, body, and tail regions of the organ. Store one half of each pancreas in neutral buffered formalin. Snap-freeze the other half of each pancreas in liquid nitrogen for separate molecular biology experiments.

1.3 After 72-hours, transfer the formalin-fixed tissues into 70% ethanol for long-term storage.

**2. Paraffin Embedding and Microtome**

2.1 Dehydrate and infiltrate formalin-fixed pancreata with wax using an automated tissue processing machine, via a series of ethanol washes ranging from 70% ethanol to 100% ethanol, incubation with xylene, and soak pancreata in Paraplast Embedding Media paraffin wax (Table 1).

**Table 1: Cassette dehydration and paraffin infiltration protocol**

| **Chemical** | **Incubation length (minutes)** | **Cycles** |
| --- | --- | --- |
| 70% ethanol | 30 | 2 |
| 80% ethanol | 30 | 2 |
| 95% ethanol | 30 | 2 |
| 100% ethanol | 25 | 2 |
| xylene | 20 | 2 |
| xylene | 45 | 1 |
| paraffin wax | 30 | 4 |

2.2 Embed pancreata in paraffin blocks using a heated paraffin embedding module and Paraplast Embedding Media. Orientate samples so that the medial edge of the organ is in the plane to be sectioned, with approximately equal portions of head, body, and tail regions.

2.3 Incubate paraffin blocks on ice for one hour immediately before sectioning.

2.4 Cut 5 µm serial sections with a Jung Biocut 2035 Microtome, set at an angle of 5°, or equivalent, using S35 fine microtome blades. Generate one hundred slides per pancreas, with one section per slide.

2.5 Transfer sections to a Superfrost Plus adhesion microscope slide; use a water bath set to 50°C to remove any wrinkles in the section and pick up one section per slide. Air-dry slides until sections are completely dry and store at room temperature until required.

2.6 Immediately before IHC, incubate slides at 55°C for 60-minutes to ensure the tissue section has fully bonded with the microscope slide.

**3. Multiplex Immunohistochemistry**

Select five tissue sections per experimental animal which are at least 100 µm apart, to undergo multiple-labelling for insulin, glucagon, and somatostatin. These markers are used to identify β-cells, α-cells, and δ-cells, respectively (1). Perform staining over multiple rounds if required; however, to control for day-to-day variation in the process, include one section per animal in each round of staining.

3.1 Deparaffinisation and rehydration of tissue sections. Using a Coplin jar, staining racks with staining dishes, or similar, perform the following incubations:

3.1.1 Two 5-minute incubations in xylene.

3.1.2 Two 2-minute incubations in decreasing concentrations of ethanol solutions (100%, 90%, 70%, 50%). Use dH2O to dilute ethanol.

3.1.3 Two 5-minute incubations in 1 x PBS.

3.2 Antigen retrieval:

3.2.1 Transfer the slides into slide racks from the 2100 Antigen Retriever and fill the slide racks with 10 mM sodium citrate buffer.

3.2.2 Place slide racks into a 2100 Antigen Retriever and perform a standard processing cycle. Allow slides to cool to room temperature in the sodium citrate buffer once the heat-pressure cycle is complete.

3.2.3 Wash slides three times in 1x PBS for 5-minutes each.

3.3 Blocking:

3.3.1 Transfer slides into 2% BSA in 1x PBS and incubate for one hour at room temperature.

Note: this blocking buffer is freshly made before use.

3.3.2 Wash slides three times in 1x PBS for 5-minutes each.

3.3.3 Blot the edges and sides of each slide with lint-free paper, and place slides horizontally in a humidity chamber.

3.3.4 Draw a line across the top and bottom borders of tissue with a hydrophobic barrier pen. Allow the hydrophobic barrier to dry. As this only takes a few seconds, the tissue section should not dry out during this time.

3.3.5 Add 170 µL of goat serum blocking buffer to each slide. Incubate slides with goat serum blocking buffer for one hour at room temperature.

3.4 Primary antibody application:

3.4.1 Pour off excess goat serum blocking buffer, and replace with 170 µL primary antibodies cocktail, with antibodies diluted in 2% BSA with 0.05% sodium azide, in 1x PBS (Table 2). This cocktail contains all three primary antibodies.

Note: these primary antibodies against insulin, glucagon, and somatostatin have been validated for use to identify endocrine cell populations in tissue sections from sheep pancreata (1-4).

3.4.2 Incubate slides in the humidity chamber at 4ºC overnight.

3.4.3 Following incubation, wash slides three times in 1x PBS with 0.1% TWEEN-20 for 5-minutes each.

**Table 2: Primary and secondary antibody dilutions for insulin, glucagon, and somatostatin multiplex immunohistochemistry**

| **Target/name** | **Raised in** | **Clonality** | **Supplier** | **Catalogue Number** | **Final Dilution** |
| --- | --- | --- | --- | --- | --- |
| Insulin | Guinea pig | Polyclonal | DAKO | DAKOA056401 | 1:500 |
| Glucagon | Mouse | Monoclonal | Sigma Aldrich | G2654.5mL | 1:250 |
| Somatostatin | Rabbit | Polyclonal | DAKO | DAKOA056601 | 1:500 |
| Anti-guinea-pig IgG (H+L) Alexa Fluor 647 | Goat | Polyclonal | Invitrogen | A-21450 | 1:400 |
| Anti-mouse IgG (H+L) Alexa Fluor 488 | Goat | Polyclonal | Invitrogen | A-11029 | 1:400 |
| Anti-rabbit IgG (H+L) Alexa Fluor 594 | Goat | Polyclonal | Invitrogen | A-11037 | 1:400 |

3.5 Secondary antibody application:

3.5.1 Add 170 µL of secondary antibody cocktail to each slide. This cocktail contains all three secondary antibodies diluted to 5 µg/mL in 1x PBS (Table 2).

3.5.2 Incubate slides for one hour at room temperature inside a humidity chamber protected from light.

3.5.3 Wash slides three times in 1x PBS with 0.1% TWEEN-20 for 5-minutes each.

3.6 DAPI staining:

3.6.1 Add 170 µL of DAPI diluted to 0.5 µg/mL in 1x PBS to each slide.

3.6.2 Incubate slides for 30-minutes at room temperature inside a humidity chamber protected from light.

3.6.3 Wash slides three times in 1x PBS with 0.1% TWEEN-20 for 5-minutes each.

3.7 Add two drops of ProLong Gold Antifade Reagent to each slide and apply a coverslip.

3.8 Seal slides with clear nail varnish, and cure at room temperature protected from light for a minimum of 48 hours before being imaged.

**References**

1. Limesand SW, Jensen J, Hutton JC, Hay WW. Diminished β-cell replication contributes to reduced β-cell mass in fetal sheep with intrauterine growth restriction. American Journal of Physiology - Regulatory, Integrative and Comparative Physiology. 2005;288:R1297-R305.

2. Leos RA, Anderson MJ, Chen X, Pugmire J, Anderson KA, Limesand SW. Chronic exposure to elevated norepinephrine suppresses insulin secretion in fetal sheep with placental insufficiency and intrauterine growth restriction. American Journal of Physiology - Endocrinology and Metabolism. 2010;298:E770-E8.

3. Rozance PJ, Anderson M, Martinez M, Fahy A, Macko AR, Kailey J, et al. Placental insufficiency decreases pancreatic vascularity and disrupts hepatocyte growth factor signaling in the pancreatic islet endothelial cell in fetal sheep. Diabetes. 2015;64:555-64.

4. Frost MS, Zehri AH, Limesand SW, Hay Jr WW, Rozance PJ. Differential effects of chronic pulsatile versus chronic constant maternal hyperglycemia on fetal pancreatic β-cells. Journal of Pregnancy. 2012;2012.
