# Supplementary material for: Whole-slide imaging and a Fiji-based image analysis workflow of immunohistochemistry staining of pancreatic islets
Source: MethodsX. 2022 Sep 13;9:101856. doi: 10.1016/j.mex.2022.101856 (PMC9531276; doi:10.1016/j.mex.2022.101856)
Supplement: Supplementary file 2 [file mmc2.docx]

// ****STEP 2: CALCULATING BACKGROUND AREA AND IMAGE ANALYSIS MACRO****

//

//

//This is the second step in the sequence of image analysis described by the protocol "Visualising pancreatic islets; a protocol for multiplex IHC staining, whole-slide imaging, and image analysis workflow

//using open source software".

//

//NOTE:

//*The hierarchy of files must be as described in the body of the protocol.

//

//*This macro has been written with the intention of analysing all slide runs through the entire project using one threshold for each image type. However, the day-to-day variation between different

//slide runs may impact the intensity of staining imaged. Therefore, although this macro has been written with the intention of analysing an entire data set from multiple slide runs at once, the option to

//instead only run the analysis on a subsection of slide runs has been included, but hashed out. If required, please use the array slices included in the macro.

//

//*These analyses will only be performed where the slideID folder has "Crop" in its name.

//

//*All outputs will be generated in a single .txt file.

//

//*The user needs to select the folder termed protocol ID (e.g. insulin, glucagon, somatostatin, and DAPI". The macro will then open up the first slide run folder (e.g. "Slide 10"), and the first slide ID folder with the word

//"Crop" at the end of its name will be opened. The macro will then proceed to measure background area, insulin positive staining area, glucagon positive staining area, and somatostatin positive staining area, for

//every image within the slide ID folder. The macro will then move onto the next slideID folder and repeat the process until all image analyses have been completed.

//

//*Confirmatory print statements have been left within the macro - these will appear in the macro log as it is being ran and are to troubleshoot any errors that may arise. These can be hashed out to speed up the macro.

//

//*Calibrating the number of pixels measured to actual size in microns must be performed and entered into the macro by the user, and entered into the macro

protocolID = getDirectory("Choose Source Directory to run the loop for - e.g. Insulin, Glucagon, Somatostatin, and DAPI"); //will also record the file path for protocolID.

sliderunnumber = getFileList(protocolID); //generates an array of files within selected protocolID folder - i.e. 01 Slide 20 etc.

setBatchMode(true); //speeds up macro.

//for- loop one

for(q=0; q<sliderunnumber.length; q++) { //for all the items in sliderunnumber array.

//if- statement one

if(matches(sliderunnumber[q], ".*[0-9]+/")){ //REGEX statement for if the current item ends in two numbers (e.g. 01 Slide 10).

showProgress(q+1, sliderunnumber.length);

//directory ID for sliderunnumber

sliderunnumberdirectorylocation = protocolID + sliderunnumber[q]; //concatenates file path generated in protocolID with the name of the current sliderunnumber item.

print(sliderunnumberdirectorylocation); //confirmatory statement.

print("this is the slide number directory location"); //confirmatory statement.

slideID = getFileList(sliderunnumberdirectorylocation); //generates an array of files within the current sliderunnumber folder - i.e. EB_160130 Sild IGS Slide 10 15S080.

print(slideID.length); //confirmatory statement.

//for- loop two

for(j=0; j<slideID.length; j++) {

print(slideID[j]); //confirmatory statement.

print("this is the slide ID currently being worked on"); //confirmatory statement.

slideIDfoldername = slideID[j];

print(slideIDfoldername); //confirmatory statement.

print("this is the slideIDfoldername of slideID"); //confirmatory statement.

isCrop = substring(slideID[j], (lengthOf (slideID[j])-5), (lengthOf (slideID[j])-1)); //this defines the part of the folder name that will contain the word "crop", if present.

//if- statement two

if(isCrop == "Crop") { //if iscrop is "Crop", then the following will be performed.

slideIDdirectorylocation = sliderunnumberdirectorylocation + slideIDfoldername; //concatenates sliderunnumberdirectorylocation with the name of the folder defined in slideIDfoldername above.

print(slideIDdirectorylocation); //will print the file directory of slideIDdirectorylocation.

slideIDdirectoryname = File.getName(slideIDdirectorylocation); //File.getName calls up the title of the folder name of slideIDdirectorylocation.

print(slideIDdirectoryname); //confirmatory statement.

outputs = slideIDdirectorylocation + "outputs"; //concatenates the file location of slideIDdirectorylocation and "outputs".

print(outputs); //confirmatory statement.

print("this is the directory location where the outputs folder will be created"); //confirmatory statement.

//creating the output directory for each slideID

File.makeDirectory(outputs); //generating the output folder.

print("outputs directory successfully generated"); //confirmatory statement.

imageID = getFileList(slideIDdirectorylocation); //generates an array list of files in slideIDdirectorylocation.

//for- loop three

for (i=0; i<imageID.length; i++) {

print(i); //confirmatory statement.

print(imageID[i]); //confirmatory statement.

imageIDbasename = substring (imageID[i], 0, (lengthOf(imageID[i])-5)); //defining the name of each FOV without the suffix of -B.tif, -G.tif, -Y.tif, or -O.tif.

imagecolour = substring (imageID[i], (lengthOf (imageID[i])-5), (lengthOf (imageID[i])-4)); //As for the above substring. Except here, index1 and index2 define the character in between the last 4th and 5th positions in list name. (i.e. Y, B, G, or O).

print(imageIDbasename); //confirmatory statement.

print("this is the imageIDbasename"); //confirmatory statement.

print(imagecolour); //confirmatory statement.

print("this is the imagecolour currently being worked on");

//if- statement three

if (imagecolour == "B") {

//define image containing DAPI staining

nucleiimage = imageIDbasename + "B.TIF"; //defines the DAPI image.

open(slideIDdirectorylocation + nucleiimage); //opens the DAPI image.

//define image containing glucagon staining

glucagonimage = imageIDbasename + "G.TIF"; //defines the glucagon image.

open(slideIDdirectorylocation + glucagonimage); //opens the glucagon image.

//define image containing somatostatin staining

somatostatinimage = imageIDbasename + "O.TIF"; //defines the somatostatin image.

open(slideIDdirectorylocation + somatostatinimage); //opens the somatostatin image.

//define image containing insulin staining

insulinimage = imageIDbasename + "Y.TIF"; //defines the insulin image.

open(slideIDdirectorylocation + insulinimage); //opens the insulin image.

//merge all four channels into one composite image

run("Merge Channels...", "c1=["+ insulinimage +"] c2=["+ glucagonimage +"] c3=["+ nucleiimage +"] c7=["+ somatostatinimage +"] create");

selectWindow("Composite"); //ensures composite image is selected.

rename(imageIDbasename + "RGB composite.tif"); //renames composite image.

saveAs("TIFF", outputs + "\\" + imageIDbasename + "RGB composite.tif"); //saves composite image to outputs folder.

print("Raw composite saved as successfully");

selectWindow(imageIDbasename + "RGB composite.tif"); //ensures composite image is selected.

run("Set Scale...", "distance=62 known=20 pixel=1 unit=um global"); //calibrates images.

run("Stack to RGB"); //converts the composite image, which has four colour layers, into one. Will add the suffix "(RGB)" to each composite image.

print("image converted to RGB successfully"); //confirmatory statement.

selectWindow(imageIDbasename + "RGB composite.tif (RGB)"); //ensures composite (RGB) image is selected.

run("8-bit"); //converts composite (RGB) image to 8-bit format.

rename(imageIDbasename + "RGB composite.TIF"); //renames composite (RGB) 8-bit image.

print("image renamed successfully");

//apply a threshold to composite (RGB) 8-bit image

selectWindow(imageIDbasename + "RGB composite.TIF"); //ensures composite (RGB) 8-bit image is selected.

setAutoThreshold("Default"); //sets threshold method.

setThreshold(0, 17); //sets image threshold. This should be checked by the user.

run("Convert to Mask"); //inverts image colours.

run("Options...", "iterations=4 count=1 black do=Close"); //defines binary options for the image, and performs the binary switch.

print("threshold applied to composite (RGB) 8-bit image successfully"); //confirmatory statement.

//measure all areas without tissue

selectWindow(imageIDbasename + "RGB composite.TIF"); //ensures composite (RGB) 8-bit image is selected.

rename(imageIDbasename + "nontissue area.TIF");

run("Set Measurements...", "area perimeter feret's area_fraction limit display add redirect=None decimal=3"); //ensures correct measurements will be performed.

run("Analyze Particles...", "size=250-Infinity show=Outlines display include summarize add"); //performs the analysis of the background of each image.

print("image measured successfully"); //confirmatory statement.

selectWindow(imageIDbasename + "nontissue area.TIF");

run("Duplicate...", " ");

rename(imageIDbasename + "RGB composite.TIF");

selectWindow(imageIDbasename + "RGB composite.TIF");

run("Create Selection");

selectWindow(imageIDbasename + "RGB composite.tif");

run("Restore Selection");

run("Flatten");

saveAs("TIFF", outputs + "\\" + imageIDbasename + "RGB composite with nontissue area ROI.TIF"); //saves a copy of the RGB composite image with all background areas measured included.

selectWindow(imageIDbasename + "nontissue area.TIF");

saveAs("TIFF", outputs + "\\" + imageIDbasename + "threshold composite with nontissue area ROI.TIF"); //saves a copy of the composite image with threshold applied with all background areas measured included.

roiManager("reset");

close("\\Others"); //closes open images.

close();

print("area with no tissue calculated"); //confirmatory statement.

}; //closing if- statement three

//if- statement four

if (imagecolour == "B") {

//measure total area for each FOV

open(slideIDdirectorylocation + nucleiimage);

rename(imageIDbasename + "total image area");

setAutoThreshold("Default dark"); //apply threshold to image.

setThreshold(1, 255); //should be checked by user.

run("Convert to Mask");

run("Fill Holes");

run("Select All");

run("Set Measurements...", "area perimeter feret's area_fraction limit display add redirect=None decimal=3"); //ensures correct measurements will be performed for each image cluster.

run("Analyze Particles...", "size=0-Infinity show=Outlines display include summarize"); //performs the analysis of the background of each image.

print("total image area measured successfully"); //confirmatory statement.

selectWindow(imageIDbasename + "total image area"); //selects total image area window.

run("Restore Selection");

run("Flatten");

selectWindow(imageIDbasename + "total image area");

saveAs("TIFF", outputs + "\\" + imageIDbasename + "total image area"); //saves a copy of the image with the total area measured.

print("image with total image area ROI drawn on, saved successfully"); //confirmatory statement.

roiManager("reset"); //resets ROI manager.

close("\\Others"); //closes all opened windows.

close();

print("total FOV area calculated"); //confirmatory statement.

}; //closing if- statement four

//if- statement five

if(imagecolour == "Y"){

//measure total area positive for insulin staining for each FOV

open(slideIDdirectorylocation + insulinimage);

rename(imageIDbasename + "insulin");

setThreshold(30, 255); //this threshold should be checked by the user for each image set.

run("Convert to Mask");

run("Set Measurements...", "area perimeter feret's area_fraction limit display add redirect=None decimal=3"); //sets measurement parameters to be performed.

run("Analyze Particles...", "size=10-Infinity show=Outlines display summarize add"); //analyses the image.

print("total area positive for insulin staining measured"); //confirmatory statement.

run("Flatten");

close();

close();

saveAs("TIFF", outputs + "\\" + imageIDbasename + "insulin.TIF"); //saves a copy of the insulin image with the positively staining region included.

print("image with total insulin area ROI drawn on, saved successfully"); //confirmatory statement.

roiManager("reset"); //resets ROI manager.

close(); //closes open image.

print("total insulin positive area calculated"); //confirmatory statement.

}; //closing if- statement five

//if- statement six

if(imagecolour == "G"){

//measure total area positive for glucagon staining for each FOV

open(slideIDdirectorylocation + glucagonimage);

rename(imageIDbasename + "glucagon");

setThreshold(20, 255); //this threshold should be checked by the user for each image set.

run("Convert to Mask");

run("Set Measurements...", "area perimeter feret's area_fraction limit display add redirect=None decimal=3"); //sets measurement parameters to be performed.

run("Analyze Particles...", "size=10-Infinity show=Outlines display summarize add"); //analyses the image.

print("total area positive for glucagon staining measured"); //confirmatory statement.

run("Flatten");

close();

close();

saveAs("TIFF", outputs + "\\" + imageIDbasename + "glucagon.TIF"); //saves a copy of the glucagon image with the positively staining region included.

print("image with total glucagon area ROI drawn on, saved successfully"); //confirmatory statement.

roiManager("reset"); //resets ROI manager.

close(); //closes open image.

print("total glucagon positive area calculated"); //confirmatory statement.

}; //closing if- statement six

//if- statement seven

if(imagecolour == "O"){

//measure total area positive for somatostatin staining for each FOV

open(slideIDdirectorylocation + somatostatinimage);

rename(imageIDbasename + "somatostatin");

setThreshold(25, 255); //this threshold should be checked by the user for each image set.

run("Convert to Mask");

run("Set Measurements...", "area perimeter feret's area_fraction limit display add redirect=None decimal=3"); //sets measurement parameters to be performed.

run("Analyze Particles...", "size=10-Infinity show=Outlines display summarize add"); //analyses the image.

print("total area positive for somatostatin staining measured"); //confirmatory statement.

run("Flatten");

close();

close();

saveAs("TIFF", outputs + "\\" + imageIDbasename + "somatostatin.TIF"); //saves a copy of the somatostatin image with the positively staining region included.

print("image with total somatostatin area ROI drawn on, saved successfully"); //confirmatory statement.

roiManager("reset"); //resets ROI manager.

close(); //closes open image.

print("total somatostatin positive area calculated"); //confirmatory statement.

}; //closing if- statement seven

}; //closing for- loop three

//saving the summary and results for each slideID

directoryname = File.getName(slideIDdirectorylocation);

slidename = substring(directoryname, 0, (lengthOf(directoryname)-5));

summarytitle = slidename + " Summary";

finalsummarytitle = "outputs\\" + summarytitle + ".txt";

print(finalsummarytitle);

resultstitle = slidename + " Results";

finalresultstitle = "outputs\\" + resultstitle + ".txt";

print(finalresultstitle);

selectWindow("Summary");

saveAs("txt", outputs + "\\" + summarytitle);

print("successfully saved summary"); //confirmatory statement.

run("Close");

print("successfully closed summary"); //confirmatory statement.

selectWindow("Results");

saveAs("txt", outputs + "\\" + resultstitle);

print("successfully saved results"); //confirmatory statement.

selectWindow("Results");

run("Close");

print("successfully closed results"); //confirmatory statement.

}; //closing if- statement two

//else-statement one

else {

print(slideIDfoldername);

print("Filename does not end in Crop");

}; //closing else- statement one

}; //closing for loop two

}; //closing if statement one

}; //closing for loop two

print("analysis completed");
