# Supplementary material for: Whole-slide imaging and a Fiji-based image analysis workflow of immunohistochemistry staining of pancreatic islets
Source: MethodsX. 2022 Sep 13;9:101856. doi: 10.1016/j.mex.2022.101856 (PMC9531276; doi:10.1016/j.mex.2022.101856)
Supplement: Supplementary file 3 [file mmc3.docx]

// ****STEP 1: CROPPING AND RENAMING MACRO****

//

//

//This is the first step in the sequence of image analysis described by the protocol "Visualising pancreatic islets; a protocol for multiplex IHC staining, whole-slide imaging, and image analysis workflow

//using open source software".

//

//NOTE:

//*The hierarchy of files must be as described in the body of the protocol.

//

//*The user needs to select the folder termed protocol ID (e.g. "insulin, glucagon, somatostatin, and DAPI". The macro will then open up the first slide run folder (e.g. "Slide 10"), and the first slide ID folder

//(e.g. "EB_160117 Sild IGS Slide 10 15S080"). The macro will then generate a new folder inside the current slide ID folder with the same folder name with "-Crop" appended to the end of the file name. Each image will

//then be opened and the specified crop will be applied. This process will be repeated for all folders within the slide run folder, and then move on to the next slide run folder in the protocol ID, and repeat the process.

//

//*We chose to move these crop folders to a new file location (keeping within the same file hierarchy as described in the body of the protocol, to ensure zero confusion.

//However, this is a individual users preference, as subsequent macros specify 'crop' files to perform further downstream actions.

//

//*Confirmatory print statements have been left within the macro - these will appear in the macro log as it is being ran and are to troubleshoot any errors that may arise. These can be hashed out to speed up the macro.

//

//*The location of where to crop was manually identified to be within the centre of each FOV where there was zero overlap with the subsequent FOV. This may need to be determined by the user.

protocolID = getDirectory("Choose Source Directory to run the loop for - e.g. Insulin, Glucagon, Somatostatin, and DAPI"); //will also record the file path for protocolID.

sliderunnumber = getFileList(protocolID); //generates an array of files within selected protocolID folder - i.e. 01 Slide 20 etc.

setBatchMode(true); //speeds up macro.

//for- loop one

for(l=0; l<sliderunnumber.length; l++){ //for all the items in sliderunnumber array.

//if- statement one

if(matches(sliderunnumber[l], ".*[0-9]+/$")){ //REGEX statement for if current item ends in two numbers (e.g. "01 Slide 10").

print(sliderunnumber[l]); //confirmatory statement.

showProgress(l+1, sliderunnumber.length);

//directory ID for sliderunnumber

sliderunnumberdirectorylocation = protocolID + sliderunnumber[l]; //concatenates file path generated in protocolID with the name of the current sliderunnumber item.

print(sliderunnumberdirectorylocation); //confirmatory statement.

print("this is the slide number directory location"); //confirmatory statement.

slideID = getFileList(sliderunnumberdirectorylocation); //generates an array of files within the current sliderunnumber folder - i.e. EB_160130 Sild IGS Slide 10 15S080.

//for- loop two

for(k=0; k<slideID.length; k++){ //for all items in sliderunnumberdirectorylocation array.

//if- statement two

if(matches(slideID[k], ".*[0-9]+/$")){ //if the current item ends in two numbers (REGEX statement).

slideIDdirectorylocation = sliderunnumberdirectorylocation + slideID[k]; //concatenates file path generated in sliderunnumberdirectorylocation with the slideID of the current item.

print(slideIDdirectorylocation); //confirmatory statement.

print("this is the current slideID directory location"); //confirmatory statement.

imageID = getFileList(slideIDdirectorylocation); //generates an array of files within the original slideIDdirectorylocation.

print(imageID.length); //confirmatory statement.

print("this is the number of files within the original imageID folder location"); //confirmatory statement.

newslideID = replace(slideID[k], "^EB_[0-9]{6} [ABC]{4} ", ""); //these two replace functions clean the new file path name.

newslideID = replace(newslideID, "/$", "");

print(newslideID); //confirmatory statement.

newslideIDdirectorylocation = sliderunnumberdirectorylocation + newslideID + " Crop"; //concatenates file path generated in sliderunnumberdirectorylocation with the newslideID, and the ending " Crop", to match with current file formats.

print(newslideIDdirectorylocation); //confirmatory statement.

File.makeDirectory(newslideIDdirectorylocation);

//for- loop three

for(i=0; i<imageID.length; i++){ //for all items in imageID array.

print(imageID[i]); //confirmatory statement to show the name of the current item.

//if-statement three

if(matches(imageID[i], ".*.TIF$")); //if the current item ends in a .TIF (REGEX statement).

open(slideIDdirectorylocation + imageID[i]); //opens the current image ID in the original slidedirectorylocation.

run("Specify...", "width=1270 height=934 x=635 y=467 centered"); //selects the specified region within the opened image. This was the correct size for our images. As such, this may need modifying by the end user.

run("Crop"); //performs cropping action.

newslideID = replace(newslideID, "^[\\s\\S]{25}", ""); //using a REGEX statement, this will shorten the newslideID generated above to remove excess characters.

newslideID = replace(newslideID, "$//", "$");

newimageID = newslideID + " " + imageID[i]; //generates a name for the newly cropped image.

saveAs("TIFF", newslideIDdirectorylocation + "\\ " + newimageID); //saves the cropped image in the newslideIDdirectorylocation generated above, with \\ to separate folder and name path, with the new image name.

close(); //closes the current image.

} //end of for- loop three

} //end of if- statement two

//if- statement three - counterpart to cropping if- statement two

if(matches(slideID[k], ".*[A-Za-z]+/$")){ //counterpart to if statement two - if the criteria in if statement two are not met, then perform the following (i.e. if the slide name ends in letters).

print("not a valid slideID for this function"); //confirmatory statement to show why this file was not actioned.

} //end of cropping if- statement three

} //end of for- loop two

} //end of if- statement one

//if- statement four - counterpart to cropping if- statement one

if(matches(sliderunnumber[l], ".*[A-Za-z]+/$")){ //counterpart to if statement one - if the criteria in if statement one are not met, then perform the following (i.e. if the sliderunnumber ends in letters).

print("not an information containing folder"); //confirmatory statement to show why this file was not actioned.

} //end of if statement four

} //end of for- loop one

print("Cropping and renaming macro is completed");
